# A Randomised, Double-Blind, Placebo-Controlled Trial of Probiotic and Postbiotic Strains in Healthy Adults with Self-Reported Anxiety: Effects on Mood, Vitality, Quality of Life and Perceived Stress

**DOI:** 10.3390/brainsci16040419

**Published:** 2026-04-16

**Authors:** Richard Day, Daniel Friedman, Ana Cardoso, Malwina Naghibi, Adria Pont, Juan Martinez-Blanch, Araceli Lamelas, Empar Chenoll, Charles Kakilla, Kieran Rea, Vineetha Vijayakumar

**Affiliations:** 1 ADM Research and Development, Medical Department, ADM Health & Wellness, London EC3R 7AG, UK; richard.day@adm.com (R.D.); malwina.naghibi@adm.com (M.N.); vineetha.vijayakumar@adm.com (V.V.); 2 ADM Research and Development Center-Biopolis, Parc Científic Universitat de València, 46980 Paterna, Spain; adria.pont@adm.com (A.P.); juan.martinezblanch@adm.com (J.M.-B.); araceli.lamelas@adm.com (A.L.); maria.chenoll@adm.com (E.C.); 3 Atlantia Food Clinical Trials, Heron House Offices, Blackpool, T23 R50R Cork, Ireland; ckakilla@atlantiatrials.com (C.K.); krea@atlantiatrials.com (K.R.)

**Keywords:** gut–brain axis, postbiotic, probiotic, stress, vitality, psychobiotic

## Abstract

**Background:** Subclinical psychological symptoms—such as low mood, perceived stress, and poor sleep—affect a large portion of the population and can impair quality of life despite remaining below clinical thresholds. The gut–brain axis has emerged as a promising target for interventions that support emotional and psychological resilience. Probiotics and postbiotics are gaining attention for their potential to modulate mood and stress via microbiome-related mechanisms, but human evidence remains limited, particularly in non-clinical populations. **Objectives**: We aimed to assess the effects of a two-strain combination of live microorganisms alongside a two-strain combination of heat-treated inactivated microorganisms on outcomes associated with anxiety, mood, perceived stress, and quality of life in healthy adults experiencing mild stress. **Methods**: This study was conducted in two parts. In Part I, a randomized, double-blind, placebo-controlled study, 100 participants were randomized to receive either a blend of live microorganisms (*Bifidobacterium longum* CECT 7347 and *Lactobacillus rhamnosus* CECT 8361) or an identical placebo once daily for 12 weeks. In Part II, a pilot feasibility study, a subset of eight placebo non-responders from Part I received the heat-inactivated preparation of the same bacterial strains in a 6-week trial extension phase. For Parts I and II, the primary outcome was the change in the Hamilton Anxiety Rating Scale (HAM-A). Secondary outcomes included measures of mood (Beck Depression Inventory (BDI); Patient Health Questionnaire-9 (PHQ-9)), stress (state and trait anxiety inventory (STAI); Perceived Stress Scale (PSS)), sleep (Pittsburgh Sleep Quality Index (PSQI)), quality of life (36-item Short Form Survey (SF-36)), gastrointestinal symptoms (Gastrointestinal Symptom Rating Scale (GSRS)), salivary cortisol and microbiome modulation. **Results**: In **Part I**, there were no significant effects of the live blend on the HAM-A, indicating that the primary endpoint was not met. In addition, no significant effects were seen on the STAI or PSS scores when compared to the placebo. However, participants consuming the live blend trended toward a reduction in total PHQ-9 scores compared to placebo (*p* = 0.089), whilst preliminary exploratory analyses suggested an improvement in anhedonia (*p* = 0.045). Furthermore, there was a significant improvement in the vitality domain of the SF-36 compared to placebo (*p* = 0.017). On microbiome analysis, it was noted that consumption of the live blend was linked to the preservation of butyrate-producing bacteria, particularly members of the *Pseudoflavonifractor* genus and the *Clostridium* SGB6179 species. Furthermore, the abundance of *B. longum* species was found to be inversely associated with the total PSS Scores. In **Part II**, supplementation with the inactivated preparation resulted in significant within-group improvements for the vitality (*p* = 0.006) and social functioning (*p* = 0.010) domains of the SF-36 and improvements in PSS scores compared to baseline (*p* = 0.050). **Conclusions**: Supplementation with either the dual-strain live or inactivated formulations was associated with significant improvements in the vitality domain of the SF-36, whilst participants receiving the inactivated formulation demonstrated lower perceived stress and improved social functioning compared to baseline. Overall, the findings from this pilot study suggest that these two biotic consortia are well-tolerated and may be associated with improvements in measures of vitality in individuals with subclinical psychological symptoms. The subtle observations detected for stress and anhedonia suggest that further well-powered trials are needed to better characterize these findings, potentially in populations with greater baseline symptomatology.

## 1. Introduction

Anxiety is highly prevalent worldwide [1,2], with subclinical or sub-threshold anxiety affecting up to twice as many individuals as clinically diagnosed cases [3]. 

Conventional management approaches, including pharmacotherapy and cognitive behavioural therapy, are often reserved for clinically diagnosed cases and are not universally effective [4]. This underscores the need for alternative, safe, and accessible interventions, particularly in individuals with emerging or subclinical presentations [5,6].

The gut–brain axis is a bi-directional communication system influencing neuroendocrine signalling, immune responses and stress-related pathways [7,8,9]. Disruption of gut microbial communities has been linked to the pathogenesis of psychiatric conditions, including anxiety and depression [10]. This has led to increasing interest in probiotics—live microorganisms that confer a health benefit when administered in adequate amounts [11] —as a potential option for mental health management.

Preclinical studies have demonstrated the beneficial effects of bacterial strains with potential probiotic properties on anxiety-like and depressive behaviours, with specific species such as *Bifidobacterium longum* and *Lactobacillus rhamnosus* shown to influence serotonergic signalling, dampen Hypothalamus–Pituitary–Adrenal (HPA) axis hyperactivity, and modulate inflammation in animal models [12,13,14]. Some human clinical studies and meta-analyses of randomized clinical trials (RCTs) have also shown modest improvements in anxiety-related outcomes; however, findings in this area remain heterogeneous, often due to small sample sizes and methodological differences [15,16].

Postbiotics—defined as preparations of inanimate microorganisms and/or their components that confer health benefits on the host [17]—represent a newer class of gut-targeted interventions, with advantages over probiotics, including improved stability and safety [17,18,19,20].

Preclinical studies indicate that heat-inactivated bacterial strains may exert anxiolytic effects [21]. For example, Robles et al. reported that a combination of heat-inactivated *Bifidobacterium longum* subsp. *longum* CECT7347 and *Lactobacillus rhamnosus* CECT8361 significantly reduced anxiety-like behaviours in *Caenorhabditis elegans* and zebrafish [22]. However, these findings have yet to be evaluated in humans.

To assess a role for probiotics and postbiotics in mood and health, we undertook the following investigations. In Part I, we investigated the effects of 12-week supplementation with a dual-strain live blend containing *B. longum* CECT7347 and *L. rhamnosus* CECT8361 on a range of mental health outcomes in healthy adults with self-reported anxiety, in a randomized, double-blind, placebo-controlled clinical study design. In Part II, we evaluated the same strain combination in its heat-treated inactivated form over 6 weeks in an extended trial design among participants who did not respond to the placebo in Part I.

The primary outcome was a change in anxiety levels, as assessed by the Hamilton Anxiety Rating Scale (HAM-A). Secondary outcomes included the Beck Depression Inventory (BDI), Patient Health Questionnaire-9 (PHQ-9), state and trait anxiety inventory (STAI), Perceived Stress Scale (PSS), Pittsburgh Sleep Quality Index (PSQI), Gastrointestinal Symptoms Rating Scale (GSRS) and the quality of life 36-item short-form survey (SF-36). Salivary cortisol levels were used as a physiological biomarker of HPA axis activity and shotgun metagenomic fecal microbiome analysis was conducted to understand the effect of the live and inactivated preparations on the gastrointestinal microbiome. This study aimed to explore the potential of both live and inactivated formulations as new strategies to support mental wellbeing in subclinical populations.

## 2. Materials and Methods

### 2.1. Study Design

This investigation was a single-centre, randomized, double-blind, placebo-controlled, parallel group pilot study conducted in two sequential parts (ClinicalTrials.gov: NCT05562752). Part I compared a dual-strain live blend with placebo for 12 weeks; Part II represents a re-randomized extension phase in a selected subgroup of participants and does not constitute a crossover design, as participants were not exposed to both treatments in a paired manner. Part II enrolled non-responders from the placebo arm of Part I into a 6-week extension with a two-strain heat-treated inactivated formulation. Given that 80% or more of the observed benefits in some mood trials can be attributed to a strong placebo response in subclinical populations [23], non-responders were defined as having a ≤50% reduction in HAM-A score at the end of the Part I intervention [24]. The study was conducted in accordance with the principles of the Declaration of Helsinki and Good Clinical Practice and approved by the Clinical Research Ethics Committee of the Cork Teaching Hospitals, Ireland (ECM3 (ff) 9 March 2023). Written informed consent was obtained from all participants at screening.

### 2.2. Participants

Part I consisted of healthy adults aged 18–65 years with mild-to-moderate self-reported anxiety (Beck Anxiety Inventory scores ≥ 8 and ≤25) [25] and no current psychiatric diagnosis. Participants were recruited via community advertising. Key exclusion criteria were: BDI-II ≥ 25 [26]; diagnosis of depression; current use of psychotropic medication; use of talking therapies, antibiotics or probiotic/postbiotic within 8 weeks; chronic inflammatory or immunological illness; and pregnancy or breastfeeding.

Part II consisted of participants from Part I that had been allocated to the placebo arm of the trial and were deemed a non-responder (defined as a ≤50% reduction in HAM-A score at the end of the Part I, compared to baseline).

### 2.3. Randomisation and Blinding

**Figure 1:**
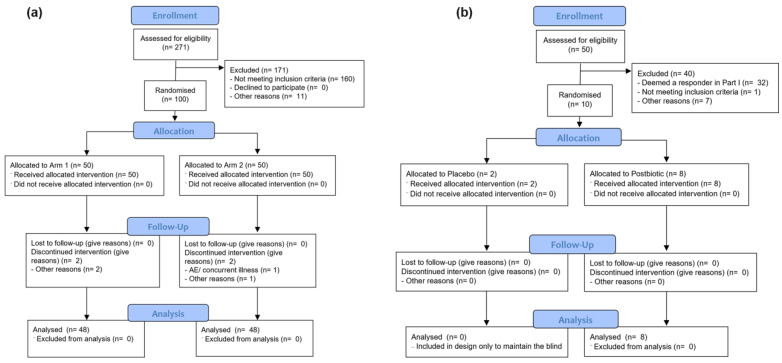
Consort diagram of (**a**) Part I, arm 1 = probiotic, arm 2 = placebo; (**b**) Part II.

An independent statistician generated an unstratified block randomisation list (1:1, concealed via a password-protected file) for Part I. Allocation concealment was ensured through sequentially labelled investigational products and sealed envelopes, preventing prediction of assignment. The randomization list was securely stored in a password-protected location with restricted access limited to the science and quality team. Both participants and study personnel remained blinded to treatment allocation throughout the study, and no deviations from the randomization procedure occurred. A total of *n* = 100 participants (*n* = 50 in the live blend arm and *n* = 50 in the placebo arm) were randomized. Two participants discontinued in the placebo group due to an adverse event (AE) or had another reason for discontinuation whilst two participants discontinued in the probiotic arm due to an AE or had another reason. Thus, 48 (48%) participants completed the study both for the placebo and probiotic groups (Intention to treat [ITT] population (Figure 1). An extra participant was excluded from the per-protocol population in the placebo group due to the intake of prohibited medicine. Investigational capsules were identical in appearance and supplied in coded bottles to maintain blinding of participants, clinicians, and data analysts. In the Part II extension, *n* = 10 eligible non-responders from the placebo arm of Part I were re-randomized 4:1 to either receive the placebo or the inactivated blend using a separate list. The randomisation in the Part II study was performed solely to maintain a blinded design. The blind was not broken until all databases were locked and analyses predefined in the Statistical Analysis Plan (SAP) were complete.

### 2.4. Interventions

In Part I of the study (N = 100), participants were randomized 1:1 to receive either a live blend or a placebo for 12 weeks. Participants (*n* = 50) consumed one capsule daily with breakfast containing a two-strain blend of *Bifidobacterium longum* CECT 7347 and *Lactobacillus rhamnosus* CECT 8361 at a concentration of 1 × 10^9^ CFU per capsule. The placebo arm (*n* = 50) received visually identical capsules containing 220 mg maltodextrin.

After a period between 21–33 weeks, 10 participants who were determined to be non-responders in the placebo arm during the first 12 weeks were invited to take part in a 6-week extension of the study for Part II. In Part II, participants were re-randomized in a 4:1 ratio to receive either an inactivated blend—a heat-treated inactivated formulation of the same *B. longum* and *L. rhamnosus* strains (*n* = 8; 1 × 10^9^ cells per capsule, or 10 mg when prepared from a inactivated formulation with a concentration of 1 × 10^11^/g)—or a matched maltodextrin (383 mg) placebo (*n* = 2). The heat-inactivated preparation and maltodextrin placebo were encapsulated in identical capsules to preserve blinding.

All participants ingested one capsule daily with breakfast for the assigned intervention period. Compliance was measured by capsule count, relative to the total number of capsules supplied. Participants with a compliance of ≥80% were deemed compliant.

### 2.5. Outcome Measures

For Part I of the trial, the primary outcome measure was change in HAM-A total score compared to the placebo. Secondary outcome measures included state and trait anxiety measured using the STAI [26], quality of life measured with the SF-36 [27], mood measured by the BDI and PHQ-9 [28], sleep measured by the PSQI [29], gastrointestinal complaints measured by the GSRS [30], perceived stress measured by the PSS [31] and physiological stress measured via salivary cortisol awakening response. As specified in the SAP, item-level testing was similarly carried out for all assessment tools described above. For cortisol saliva measurements, saliva samples were collected on waking (T0) and 30, 45 and 60 min after waking on the morning of the visits at baseline and at weeks 6 and 12. Oral hygiene procedures and eating/drinking were only permitted once all samples had been collected. As an exploratory outcome, stool samples were collected at weeks 0 and 12 to evaluate changes in the gut microbiome following consumption of the live strain blend. HAM-A scorings were assessed at weeks 0, 6 and 12 whilst all other outcomes were measured at weeks 0, 4, 6 and 12.

For Part II of the trial, HAM-A, STAI, SF-36, BDI, PHQ-9, PSQI, GSRS, PSS, salivary cortisol awakening response and stool samples were measured at weeks 0 and 6.

All questionnaires were administered by qualified psychologists in private rooms; electronic case-report forms were verified against source data at each visit. Adverse events (AEs) were coded per MedDRA v25.0 and reviewed by an independent safety monitor.

### 2.6. Sample Size Considerations

As an exploratory study, 50 participants per arm (total 100) were assessed to provide preliminary effect-size estimates for future power calculations. Ninety-six participants were retained for ITT analysis (48 per group), exceeding the minimum recommended sample size for pilot studies [27]. Ten non-responders proceeded to Part II (eight received the inactivated preparation and these eight are included in the extended trialanalysis).

### 2.7. Fecal Sample Collection and DNA Extractions

Post-collection, samples were stored and kept at −20 °C, whereupon they were transferred and analyzed at ADM-BIOPOLIS, Valencia, Spain. Fecal DNA was extracted by mechanical disruption using a FastPrep-24™ instrument (MP Biomedicals, Santa Ana, CA, USA) and subsequently isolated using a QIAamp^®^PowerFecal^®^Pro DNA-kit (QIAGEN^®^, Hilden, Germany). Finally, DNA preparation was subjected to quality control on a TapeStation™ 4200 equipment (Agilent, Santa Clara, CA, USA).

### 2.8. Fecal DNA Metagenomic Shotgun Sequencing

Fecal DNA was quantified using a Qubit Fluorometer (Thermo Fisher Scientific, Carlsbad, CA, USA). Sequencing libraries were prepared with an Illumina DNA Prep Library kit (Illumina, San Diego, CA, USA) following the manufacturer’s instructions. Once the libraries were prepared, they were quantified using the PicoGreen assay (Invitrogen, Carlsbad, CA, USA) and subsequently pooled at equimolar concentrations. The samples were sequenced using a NovaSeq 6000 platform with paired end reads of 151 base pairs. The Bcl2fastq 2.20 programme was used to translate the sequencing reads from bcl (Base Calling) to FASTQ format.

### 2.9. Bioinformatic Analysis

Optical duplicates were removed using the Clumpify tool from the BBTools suite [28] (Bushnell). Reads with a Phred quality score < Q20 and length of <50 nucleotides were filtered out using the program BBMap v38.36 from BBTools. Human genome presence was filtered by applying NGLess v1.0.0-Linux64 [29]. The Homo Sapiens hg19 genome, which is built into NGLess, was used. Subsequently, sequences were aligned to the genomes and those with alignments of >45 bases and 97% similarity were discarded. After quality control, the average number of sequences per sample was 28.5 million, with a standard deviation of 10.8 million. The number of reads were then standardized to 20 million to reduce sequencing bias using Seqtk v1.4 [30]. The remaining sequences were termed ‘high-quality sequences’ and were meant to be the final sequences.

Taxonomy profiles were constructed through Metaphlan (v4.1) [31], where the reads were aligned against single-copy genetic markers. From these alignments, the estimated number of reads contributed by a given clade for each identified taxon was obtained computationally.

Gene abundance profiling was performed using the 9.9 million gene integrated reference catalogue of the human microbiome [32], enriched with 5.5 genes from other constructed metagenomes from our studies. Those metagenomes were assembled from the ‘high-quality sequences’ using the MEGAHIT genome assembler (v3.13.0) [33]. Contigs larger than 500 bp were used to predict genes using Prodigal (v2.6.3) [34]. Filtered high-quality reads were mapped with an identity threshold of 95% to the 15.4 million gene catalogue enriched by assembled metagenomes, using Salmon v1.10.3 [35], obtaining a matrix with the counts for each gene on each sample. All the database genes were annotated using web server GhostKoala v3.1 [36]. CAZy enzymes were annotated using dbCAN3 v4.1.4 [37]. Genes were selected according to the following conditions: containing at least 10 counts in at least 10% of the samples, and having a KEGG annotation included in prokaryotic pathways.

### 2.10. Statistical Analysis

Analyses were executed in SPSS v28 following the locked SAP and an addendum approved before unblinding of Part II. Normality of the data was checked via Shapiro–Wilk’s test. For each endpoint variable, an ANCOVA (with baseline values as covariates) was used to determine statistically significant changes from baseline to week 12 between groups, where week 12 is the dependent variable, the group is the independent variable and the week 0 value is the covariate. Repeated-Measures (RMs) ANOVA was used to assess between subject differences (fixed effects: group, time, and group × time) within each group. For non-normalized data distributions, Quade’s rank ANCOVA or Friedman tests were used. For sensitivity analyses for primary and secondary endpoints where more than one time point is included, Bonferroni adjustment was applied to correct for multiple comparisons in the model. Paired *t*-tests or the non-parametric alternative, the Wilcoxon signed-rank test, were used to assess within-group data comparisons from baseline.

For Part II, data normality was assessed via Shapiro–Wilk’s test. Where the assumption of normality was met, mixed effects RM ANOVA (within subject factor) was used. For non-normalized data distributions, Friedman tests with Bonferroni-adjusted pairwise Wilcoxon signed-rank post hoc comparisons were used. Sensitivity analyses employed ANCOVA, controlling for baseline variance. The data from the two participants who received a placebo in both Part I and Part II were discarded as they were included solely to preserve blinding; only data from the eight participants taking the inactivated preparation were kept for analysis. Statistical significance was set at *p* ≤ 0.05. Consistent with American Statistical Association guidance that *p*-values should be interpreted on a continuum rather than by strict cut-offs, values between 0.05 and 0.10 were described as indicating weak evidence or a trend toward an effect rather than conclusive significance [38].

### 2.11. Gut Microbiome Statistical Analysis

Statistical analysis on gut microbiome data was conducted using samples from participants with both time points available. Data were normalized with the rarefaction technique from Phyloseq R package v1.46.0 [39] in order to perform alpha diversity analysis at both species taxonomic and gene levels. Shannon, Simpson, and Richness indices were calculated using the vegan R package v2.6-6.1 [40] and the Wilcoxon test was used to find significant differences in alpha diversity between groups. The Bray–Curtis dissimilarity matrix and PERMANOVA analysis for beta diversity on species and gene level were performed using the vegan R package, after normalization by relative frequency for each sample.

Differentially abundant taxa and genes on the different comparisons were identified by applying DESeq2 v1.42.0 [41]. The model’s formula was group + time + group:time, and a subject was included as fixed effect, as explained in the DESeq2 vignette. Raw counts were given to DESeq2, which performed ‘Relative Log Expression’ normalization including the ‘poscounts’ parameter in the ‘estimateSizeFactors’ function for handling samples with a high proportion of zeros. A taxon was considered differentially abundant if the corrected *p*-value < 0.05 and if it was present in at least 50% of the samples of one of the compared groups. A gene set enrichment analysis (GSEA) was conducted using the fgsea package in R v1.16 [42] on KEGG modules. The KEGG database was enlarged with custom modules mostly related to the gut–brain axis. Genes were pre-ranked according to the resulting statistics from DESeq2 analysis for each comparison in order to perform the GSEA. A module was differentially enriched if the adjusted *p*-value was lower than 0.05 and if the absolute value of the Normalized Enrichment Score was higher than 1.5. Only KEGG modules with all necessary genes present in the dataset were depicted in the heatmap.

Correlations between microbial abundances and clinical measures, including all the studied mental health questionnaire scores, were tested by applying MaAslin2 v1.18.0 [43]. The abundance of each bacterium was modelled including each clinical variable as the fixed effect and the subject variable as a random effect, correcting the *p*-value by the multiple bacteria tested. The normalization method selected was the same as carried out in DESeq2. Only the taxa present in >20% of the samples were considered and a correlation was significant if the adjusted *p*-value was lower than 0.05. Complex heatmap R package v2.18.0 [44] was used to construct summary heatmaps for correlations, GSEA and taxa differential abundance analysis.

## 3. Results

### 3.1. Demographic and Other Baseline Characteristics

A total of 96 participants were randomized and included in the ITT analysis, with *n* = 48 in each arm in the live blend (hereafter referred to as probiotic) and placebo groups. Groups were comparable in age (*p* = 0.714), and no statistically significant differences were observed in most demographic or lifestyle characteristics at baseline (Table 1). Participants in the probiotic group had a higher average BMI and greater total body weight at baseline compared to the placebo group. Psychological and gastrointestinal baseline scores showed no statistically significant between-group differences. One baseline trend worth noting was on the STAI-State scale, where the probiotic group reported slightly lower scores with borderline significance (*p* = 0.051), suggesting slightly lower baseline state anxiety. As for the Part II trial, most participants were female, white Irish, non-smokers and 75% consumed alcohol (Table 2).

### 3.2. Tolerability and Compliance

All participants in both parts of the trial were ≥80% compliant. In the probiotic arm, 22 AEs were reported. All AEs were of mild or moderate intensity and the most common AE was increased blood pressure (*n* = 5), which was deemed not related to the study product. There were eight (probiotic blend = 2; placebo = 6) AEs deemed related to study product. These were mostly gastrointestinal (e.g., abdominal distension, diarrhea, gastrointestinal disorder, constipation and flatulence) and were of mild severity. There was one withdrawal due to an AE in the placebo group, where the participant experienced a rash of moderate severity eight days after beginning treatment. The AE was deemed related to the placebo product and the participant was withdrawn from the study. No AEs were reported for the inactivated (hereafter referred to as postbiotic) blend. In conclusion, the pro- and postbiotics of this two strain blend were found to be well tolerated in this study.

### 3.3. Mental Health Parameters

#### 3.3.1. Anxiety Outcomes (HAM-A, STAI-Trait, STAI-State, PSS)

**Figure 2:**
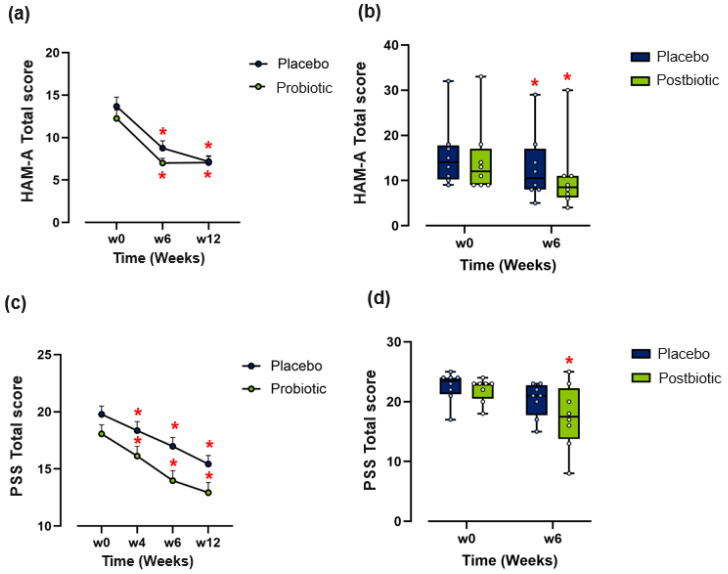
Assessment of anxiety symptoms in participants from Part I and Part II. (**a**) Part I change in HAM-A scores over 12 weeks (*n* = 48). (**b**) Part II change in HAM-A scores over 6 weeks (*n* = 8). (**c**) Part I total perceived stress scores (PSSs) over 12 weeks (*n* = 48). (**d**) Part II PSSs over 6 weeks (*n* = 8). Data points in (**a**,**c**) represent mean + SEM. ANCOVA or Friedman tests with Bonferroni post hoc analysis were used to assess within-subject differences. Paired *t*-tests or related-samples Wilcoxon signed-rank tests were used to assess within-group differences. (*) within-group significant difference from baseline (*p* ≤ 0.05).

**In Part I**, there was a significant reduction in HAM-A scores from baseline to week 12 in both the placebo and probiotic blend groups (*p* < 0.001). After controlling for baseline HAM-A scores, there was no statistically significant difference in week 12 HAM-A scores between the placebo and probiotic groups (Supplementary Table S1), suggesting that the strong placebo response limited the ability to detect between-group differences (Figure 2a). Similarly, there was a strong placebo effect in both STAI-Trait and State anxiety scores, which significantly decreased over time in both the probiotic and placebo groups and may potentially have masked any positive readouts of the probiotic blend, as between-group differences were determined to be non-significant (Supplementary Table S1). PSS scores significantly decreased in both the probiotic and placebo groups from baseline (Figure 2c; *p* < 0.001), with no significant interaction effects (Supplementary Table S1).

**In Part II**, no significant differences in week 6 HAM-A scores between the placebo and postbiotic groups were detected (Supplementary Table S2), with both groups showing significant reductions (postbiotic *p* = 0.02; placebo *p* = 0.01) in HAM-A scores from baseline (Figure 2b). However, among postbiotic-treated participants, 25% (2/8) achieved a ≥50% reduction in HAM-A scores and 62.5% (5/8) improved more during the postbiotic phase than during prior placebo treatment (Supplementary Figure S1a), although this observation should be interpreted cautiously given the comparison is likely confounded by the selection of placebo non-responders from Part I.

Using the cut-off of HAM-A ≥14 to denote clinically significant anxiety, results showed that at week 6, 87.5% (7/8) of participants in the postbiotic group had HAM-A scores below the cut-off, compared to 62.5% (5/8) in the placebo group (Supplementary Figure S1b). Interestingly, a higher proportion of participants in the postbiotic group improved their HAM-A severity category (62.5%) compared to placebo (37.5%; Supplementary Figure S1c). Whilst underpowered to detect definitive between-group differences, these results indicate a potential association with anxiety reduction following postbiotic intervention, in a subclinical population.

STAI-Trait and STAI-State scores remained similar between the groups at week 6 and when compared to baseline scores (Supplementary Table S2). Intriguingly, a significant reduction in PSS scores was detected in the postbiotic group at week 6 compared to baseline (*p* = 0.050) (Figure 2d); however, between-group differences were not significant (*p* = 0.404; Supplementary Table S2).

#### 3.3.2. Salivary Cortisol (C_max_)

In **Part I**, salivary cortisol levels in the probiotic group remained stable, while the placebo group declined significantly compared to baseline (*p* = 0.002), with a significant group × time interaction (Supplementary Table S1; *p* = 0.039). In **Part II**, whilst minor fluctuations were detected in both groups, no significant changes or trends were observed over 6 weeks.

Whilst the data in Part II were likely underpowered to detect meaningful trends over time, data from Part I suggest that while the placebo group tended to show a decline in salivary cortisol over time, no changes were detected in the C_max_ values in the probiotic group.

#### 3.3.3. Mood Outcomes (BDI, PHQ-9)

**Figure 3:**
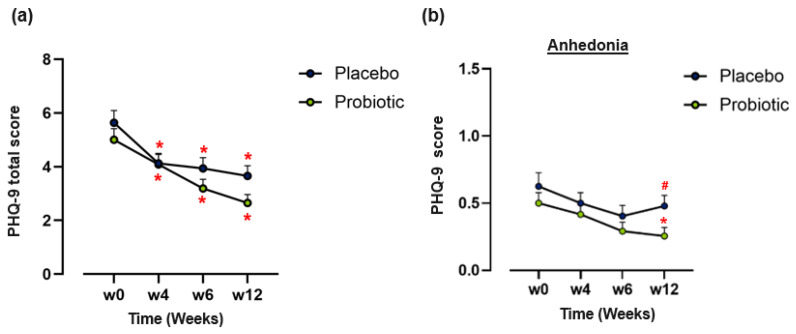
Analysis of mood symptoms in Part I of the study. (**a**) Change in Patient Health Questionnaire-9 (PHQ9) total scores over 12 weeks. (*n* = 48) (**b**) Change in PHQ9 scores for item 1 (anhedonia) over 12 weeks (*n* = 48). Data points represent mean + SEM. ANCOVA or paired *t*-tests were used to assess both between- and within-group differences, respectively. (*) Within-group significant difference from baseline (*p* ≤ 0.05). (#) Between-group significant difference (*p* ≤ 0.05).

In **Part I**, both BDI and PHQ-9 scores declined significantly in the probiotic and placebo groups, compared to baseline (*p* < 0.001). Between-group comparison of BDI scores failed to reach statistical significance (Supplementary Table S1; *p* = 0.274); however, there was a non-significant trend favouring the probiotic over placebo group for PHQ-9 total score at week 12 (*p* = 0.089) (Figure 3a).

Exploratory post hoc analysis of individual PHQ-9 items revealed a lower score in anhedonia (Item 1 of the PHQ-9) at week 12 in the probiotic group compared to placebo (mean difference: −0.26; CI −0.42, −0.10; *p* = 0.045; Figure 3b). No significant between-group differences were observed for other items of the PHQ-9 (). For **Part II,** no within- or between-group differences were detected for either the placebo or postbiotic groups for both BDI and PHQ-9 scores (Supplementary Table S2).

### 3.4. Sleep (PSQI)

In **Part I**, sleep quality improved modestly in both groups, with no significant between-group differences observed in PSQI total scores (Supplementary Table S1). A trend for a reduction in sleep medication use was observed in the probiotic group compared to placebo (*p* = 0.067) (Supplementary Figure S2a). In **Part II**, whilst greater reductions in PSQI total scores at week 6 were observed in the postbiotic group compared to placebo, these reductions were not statistically significant, likely due to the small sample size (Supplementary Table S2).

### 3.5. Quality of Life (SF-36)

**Figure 4:**
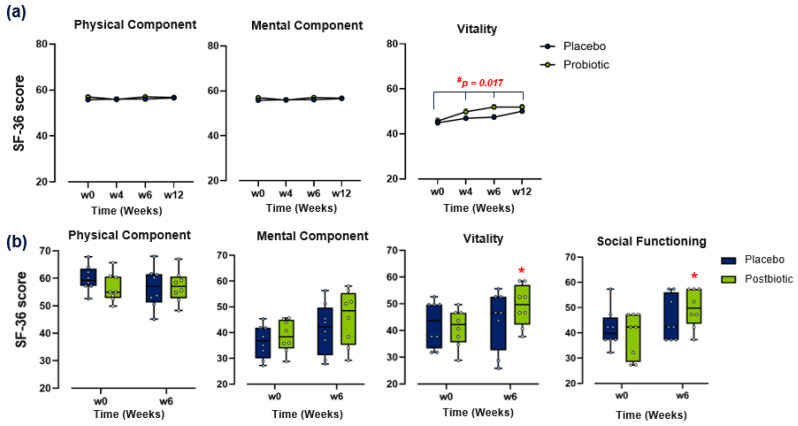
QoL subdomain score assessments in study trial Part I and II. (**a**) Change in the 36-item Short-Form health Survey (SF-36) physical component (**left** panel), mental component (**middle** panel) and vitality (**right** panel) scores over 12 weeks (*n* = 48). Data points represent mean + SEM. (**b**) Change in SF-36 physical component (**left** panel), mental component (**middle left**), vitality (**middle right**) and social functioning (**right** panel) scores over 6 weeks (*n* = 8). ANCOVA, RM ANOVA or Friedman tests with Bonferroni post hoc analysis were used to assess between-group differences. Paired *t*-tests or related-samples Wilcoxon signed-rank tests were used to assess within-group differences. (*) Within-group significant difference from baseline (*p* ≤ 0.05). (#) Between-group significant difference (*p* ≤ 0.05).

In **Part I,** quality of life, as assessed by the SF-36, showed improvements over time across both the probiotic and placebo groups, but with no significant differences between groups for the total SF-36 score. No differences in scores were detected for both the Physical and Mental Component Summary scores. However, when assessing the individual domains of the SF-36, a significant group × time interaction emerged for the vitality domain (Supplementary Table S1; *p* = 0.017;), indicating a greater improvement in the probiotic group over time, compared to placebo. (Figure 4a). The effect on SF-36 vitality appeared to peak at intermediate time points and attenuated by week 12, yielding a modest between-group difference at the study’s endpoint. Although this difference is below commonly cited minimum clinically important difference thresholds (3–5 points), these thresholds are context-dependent and primarily derived from clinical populations. The significant group × time interaction indicates that the intervention influenced the trajectory of change over time, suggesting a time-dependent effect. These findings suggest that although overall quality of life improved similarly in both groups, the probiotic blend may confer a modest additional benefit in vitality scores over time, reflecting aspects of energy and fatigue.

In **Part II**, there were no significant between-group differences for the Physical or Mental Component Summary scores of the SF-36. However, for the Mental Component Summary scores, there was a trend toward improvement in the postbiotic group, compared to baseline (*p* = 0.059). Assessment of mental health domains revealed a significantly greater effect on the vitality domain over time in the postbiotic group compared to baseline (*p* = 0.006), with a trend for improved scores compared to placebo (*p* = 0.087). A greater improvement in the social functioning score was also detected in the postbiotic group at week 6 compared to baseline (*p* = 0.010) (Supplementary Table S2 and Figure 4b).

These changes can be seen as a proxy for improved mental resilience, often linked with better mood, reduced stress, or enhanced quality of life—particularly relevant when evaluating interventions aimed at the gut–brain axis. These data indicate psychological and emotional benefits across both studies, particularly in relation to “vitality” as measured by the SF-36.

### 3.6. Gastrointestinal Symptoms (GSRS)

In **Part I**, GSRS total scores showed small reductions in both groups with no between-group differences (Supplementary Table S1). Within-subject improvements included reduced indigestion, abdominal pain and diarrhea in the probiotic group at week 6 (Supplementary Figure S2b).

In **Part II**, GSRS total scores showed small reductions in both groups with no between- or within-group differences observed (Supplementary Table S2).

### 3.7. Gut Microbiome Analysis

In **Part I**, subjects who did not have fecal samples collected at both time points were discarded from the analysis, resulting in 44 from the placebo group and 47 from the probiotic group. In **Part II**, microbiome analysis was not performed as the number of samples was too low to obtain enough statistical power to differentiate between the analyzed groups.

#### 3.7.1. Microbiome Composition

A total of 1777 different species were detected by Metaphlan v4.1 across the 182 samples. Overall, the population had a microbiome mainly dominated by the genera *Blautia* (9.77 ± 6.74%), *Bifidobacterium* (6.37 ± 8.62%), *Faecalibacterium* (6.17 ± 3.88%), *Bacteroides* (5.79 ± 5.32%) and *Phocaeicola* (5.48 ± 4.23%) (Supplementary Figure S3a), and the species *Faecalibacterium prausnitzii* (5.07 ± 3.23%), *Blautia wexlerae* (4.95 ± 4.73%), *Bifidobacterium adolescentis* (3.76 ± 5.87%), *Ruminococcus bromii* (3.73 ± 3.73%) and *Eubacterium rectale* (3.69 ± 4.42%) (Supplementary Figure S3b).

#### 3.7.2. Taxonomy Diversity Analysis

To investigate the potential mechanisms through which intervention influences the gut microbiome, metagenomic profiling was conducted on samples collected at all study visits. The Wilcoxon test showed no significant differences according to alpha diversity measures, when comparing between time points during placebo or probiotic intake (Supplementary Figure S4a). To determine the relative contribution of interventions in shaping gut microbiota, we performed principal coordinate analysis (PCoA) using the Bray–Curtis distances between the samples. The analysis revealed no distinct clustering by either group or time points (Supplementary Figure S4b). Permutational multivariate analysis of variance (PERMANOVA) indicated that the interventions significantly modified the global bacterial community although it explained very few of the total variation [R^2^ = 0.0077, (*p* = 0.001)], while most of the variation was explained by the individual effect [R^2^ = 0.8062, (*p* = 0.001)].

**Figure 5:**
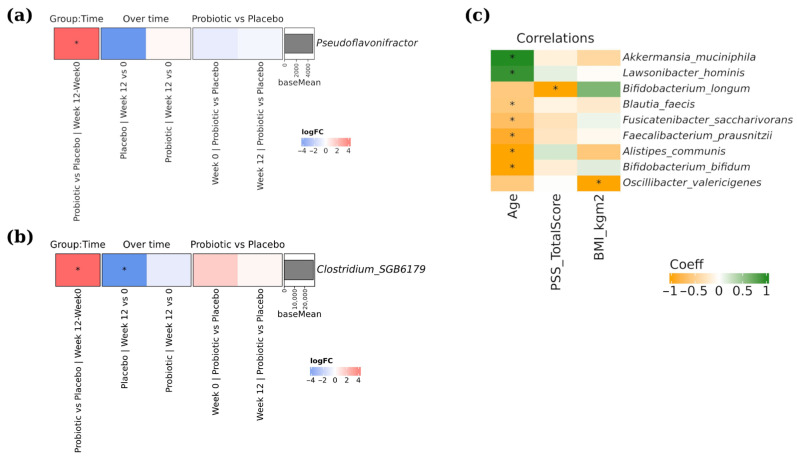
Differentially modified taxa between probiotic and placebo arms and correlations between microbial composition and clinical measures including psychometric scores. (**a**,**b**) show complex heatmaps of changes in taxon abundance (Log2FC): the first column represents the group × time interaction, the second and third columns show time comparisons within each group, and the fourth and fifth columns show group comparisons at each time point. Red indicates taxa more abundant in the first group, and blue indicates higher abundance in the second. The accompanying bar plot displays the mean normalized abundance (baseMean) of each species. Statistical significance was assessed using the DESeq2 package (Wald test), with * indicating adjusted *p* < 0.05 and presence of the taxon in at least 50% of samples in one of the compared groups. (**c**) Heatmap of correlations between species abundances and clinical measures (including mental health questionnaire scores), where green indicates positive and orange negative correlations. Only species with a significant correlation with at least one parameter are shown (* adj. *p* < 0.05).

Few taxa modified their abundance over time when placebo and probiotic supplementation were compared. Significant changes were observed in *Pseudoflavonifractor* (group × time interaction, adj. *p* = 0.0413, LogFC = 5.11), with the placebo group exhibiting downward trends over time (Figure 5a). Moreover, a significant decrease in the placebo group was observed for *Clostridium* SGB6179 species abundance (adj. *p* < 0.001, LogFC = −5.79) (Figure 5b). No changes were observed in *Pseudoflavonifractor* or *Clostridium* SGB6179 species abundance in the probiotic group. We next investigated whether there was a relationship between taxa and clinical data, modelling the abundance of the different bacteria with each clinical variable, including mental health scores, and correcting the *p*-value by the multiple bacteria tested. We identified an inverse correlation (adj. *p* = 0.0063, Coeff = −1.31) between the *Bifidobacterium longum* species abundance with the total PSS score (Figure 5c).

#### 3.7.3. Functional Profiling of Gut Microbiota

To investigate the potential microbial pathways involved in the interventions, we performed a gene set enrichment analysis (GSEA) at the module level.

**Figure 6:**
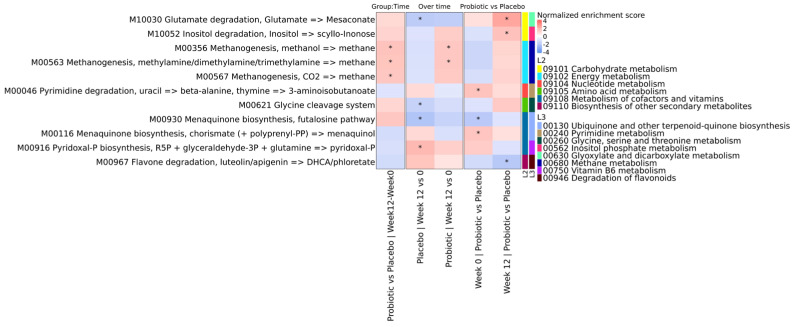
Gene set enrichment analysis of KEGG modules for fecal microbial composition. Heatmaps showing the Normalized Enrichment Score (NES) resulting from group–time interaction in the first column, between-time comparisons per group in the second and third columns, and between-group comparisons at each time in the fourth and fifth columns, on KEGG modules. Red shading indicates positive NES values, meaning the module is enriched in the first group of the comparison. Blue shading indicates negative NES values, meaning the module is enriched in the second group of the comparison. Legend: “L2” refers to KEGG annotation at general category level, while “L3” refers to KEGG annotation at pathway level. Only complete modules are depicted. Statistical significance was tested in R with the “fgsea” package by false discovery rate. * adj. *p* < 0.05.

In the placebo group, genes linked to mesaconate biosynthesis from glutamate showed a significant decline over time (adj. *p*-value = 0.0177, NES = −1.8850), leading to a higher abundance in the probiotic group at the final time point (adj. *p* < 0.001, NES = 2.43). A similar pattern was observed in genes involved in menaquinone (vitamin K2) synthesis (M00930; adj. *p* < 0.001, NES = −2.15) and glycine cleavage (M00621; adj. *p* < 0.001, NES = −1.64). Conversely, the Pyridoxal-P synthesis module (M00916) increased over time in the placebo group (adj. *p* < 0.001, NES = 1.91) whilst no significant changes were detected in the probiotic group. Moreover, genes related to methanogenesis (M00356 and M00563) increased over time during probiotic intake (adj. *p* = 0.0283, NES = 1.53; adj. *p* = 0.0283, NES = 1.54, respectively), with no significant changes detected in the placebo group (adj. *p*-values > 0.05) (Figure 6).

## 4. Discussion

This two-part, randomized, double-blind, placebo-controlled trial evaluated the effects of both a live two-strain probiotic blend and a heat-treated postbiotic blend of the same bacterial strains on psychological and somatic outcomes in healthy adults with self-reported anxiety. Both the probiotic and postbiotic blends were well-tolerated, with no serious adverse events and minor gastrointestinal complaints reported in a small number of participants. Although neither the probiotic or postbiotic outperformed the placebo on the primary outcome of anxiety (HAM-A), the study identified several effects across mood, vitality, perceived stress and the gut microbiome that contribute new evidence to the emerging field of psychobiotics and postbiotics.

### 4.1. Interpretation of Main Findings

In **Part I**, both the probiotic and placebo groups showed substantial reductions in anxiety, stress and depressive symptoms, highlighting the strong placebo responses typical in subclinical emotional wellbeing studies. These findings highlight the powerful placebo effects in psychological interventions involving subclinical populations. This has been well documented in the literature, where causal effects of placebo treatment have been demonstrated on measures typically used as primary disease endpoints, such as in trials assessing chronic pain [45,46] or depression [47,48]. Structured participation, daily routines, and repeated self-monitoring likely contribute to symptom improvement in all groups. These findings are consistent with estimates that placebo responses may account for 80% or more of the observed benefit in some mood trials [49]. Against this background, post hoc exploratory analyses suggested a signal of greater reduction in anhedonia (PHQ-9 item 1) in the probiotic group and a significant group × time effect on vitality, indicating enhanced energy, motivation and mental resilience relative to the placebo.

Both *B. longum* CECT 7347 and *L. rhamnosus* CECT 8361 have been shown to reduce anxiety-like behaviour in animal models [12,13,14,50] and, consistently, we detected an inverse correlation between the abundance of *B. longum* species and PSS scores, suggesting that the higher levels of *B. longum* could be linked to lower perceived stress in humans. The absence of a demonstrable effect on anxiety symptoms in this study, however, may reflect any number of the realities of translating pre-clinical research into clinical success, including insufficient pathology in the target population, floor effects of the tools used and challenges in scaling dosing regimens from zebrafish to humans. Of note, a 6-week supplementation with *B. longum* NCC3001 in healthy adults significantly improved PSS scores [51], although differences in probiotic dosages and lower baseline PSS scores in this study may have accounted for the lack of effect observed. Indeed, in healthy populations, the challenge in observing a reduction in stress following probiotic intervention has previously been demonstrated [52,53]. Thus, the absence of an effect may reflect below-threshold symptom baseline scores at which beneficial outcomes fail to be detected.

In contrast, exploratory analyses indicated an improvement in the PHQ-9 item 1 (anhedonia) in the probiotic group, although this finding requires cautious interpretation. Given the low baseline PHQ-9 scores in a healthy population, the scope for measurable improvement was limited (floor effects), which may partly explain the modest effect size observed for anhedonia, the clinical relevance of which remains uncertain. However, reductions in anhedonia may be particularly relevant, as this symptom has been proposed to reflect alterations in reward-related signalling networks, including dopaminergic and serotonergic pathways which are influenced by gut–brain axis communication [54]. Notably, *Bifidobacterium* strains have been shown to synthesize γ-aminobutyric acid (GABA) [55], a key neurotransmitter which may act on these signalling networks via gut–brain axis pathways. We observed a trending improvement in total PHQ-9 scores for the probiotic group which is particularly striking, given that the participants presented with mean baseline depression scores classed as ‘mild’ (<6), where beneficial effects may have gone undetected. Previous studies found significant benefits of probiotics on depression scores in people both with Major Depressive Disorder [23,56] and in populations with subclinical mood scores [57], suggesting that probiotics may have a more pronounced impact on improving low mood than anxiety. Indeed, a recent systematic meta-analyses review shows larger effect sizes of probiotics for improving depression compared to anxiety symptoms [15].

Vitality is a latent variable that comprises physical energy, wellbeing, regulation of mood, interest in life and may be involved in the counter-regulation of negative emotions [58]. To our knowledge, improvements in vitality have only been reported for one other probiotic strain. Specifically, consumption of the related strain *B. longum* 1714 for 8 weeks in healthy participants with mild sleep impairment resulted in improved vitality scores, compared to placebo [59]. Whilst *B. longum* 1714 has primarily been recognized for its effects on the HPA axis [60,61], the related strain *B. longum* CECT 7347 has traditionally been studied for its role in regulating gastrointestinal inflammation and immune health [60,62]. Our findings identify a potential role for *B. longum* CECT 7347 in modulating the HPA axis, supporting further research in exploring its effects on mental health and wellbeing.

Assessment of salivary cortisol showed that levels remained stable in participants consuming the live probiotic blend, whereas the placebo group exhibited a significant decline over time. These findings suggest a potential association between probiotic supplementation and maintenance of a more stable cortisol awakening response (C_max_), a pattern that has been linked to stress resilience, cognitive performance and immune regulation. In contrast, blunted or dysregulated cortisol profiles have been associated with adverse mood and sleep outcomes.

In parallel, microbiome analysis revealed temporal differences in the microbial composition between groups. The relative abundances of *Pseudoflavonifractor* genus and *Clostridium* SGB6179 species in the placebo group decreased or showed a decreasing trend over time, whilst levels remained stable in the probiotic group. Members of the *Pseudoflavonifractor* genus are known butyrate producers, similar to several species within the *Clostridium* genus. Previous studies have demonstrated that the increase in butyrate producers by probiotic supplementation can reduce anxiety-like behaviour in mice [63] and that a lower abundance of butyrate-producing bacteria is associated with higher anxiety levels in humans [64].

At the functional level, changes in microbial gene pathways were also observed. Genes involved in methanogenesis increased over time in the probiotic group, suggesting a potential neuroprotective role. Methane has been shown to mitigate the progression of traumatic nervous system diseases [65] and ameliorate depressive-like behaviours in rats subjected to chronic mild stress [66]. Conversely, genes associated with mesaconate synthesis from glutamate and menaquinone synthesis decreased their abundance in the placebo group over the time. Mesaconate has been implicated in counteracting neuroinflammation induced by lipopolysaccharides [67], whereas menaquinone (vitamin K2) has demonstrated anxiolytic and antidepressant effects in animal models, including rats with metabolic syndrome [68], cerebral injury [69], and natural ageing [70].

Conversely, genes related to Pyridoxal-P synthesis, also known as vitamin B6, increased their abundance over time in the placebo group but remained unchanged in the probiotic group. Vitamin B6 is an essential cofactor for the synthesis of GABA, serotonin and dopamine, and high-dose supplementation has been linked to anxiety reduction [71]. However, in the absence of direct host-level biomarkers (inflammatory or neurotrophic factors), in this study the functional relevance of these microbial gene shifts to host neurochemistry cannot be directly inferred and should rather be considered a plausible hypothesis. Future studies combining microbial functional data with host neurobiological measures are needed to clarify these relationships.

In **Part II**, the postbiotic blend produced consistent improvements across multiple psychological domains in a cohort of placebo non-responders. Although the small sample size precluded powered between-group comparisons, most participants showed greater reductions in anxiety during the postbiotic phase than during their prior placebo exposure, alongside significant within-group decreases in perceived stress and marked gains in vitality. Postbiotics have previously demonstrated improvements in anxiety symptoms in animal models [22,72] and in an irritable bowel syndrome-diagnosed population [62]. Our findings are notable given that this was a healthy population of placebo non-responders and suggest that the postbiotic may offer targeted benefits in individuals with subclinical anxiety-related symptoms.

Together, these findings highlight modest improvements in mood and anxiety; specifically, psychophysiological domains—including vitality, stress, and social functioning—may benefit more from targeted biotic interventions, particularly in placebo non-responders or those with higher symptom burden.

### 4.2. Limitations and Future Directions

Limitations include the small sample size in Part II, limited power for detecting interaction effects, and lack of biological markers (e.g., cytokines and BDNF) to clarify mechanisms. Baseline mismatches between study parts may have introduced bias and floor effects in the assessments used to measure depression and anxiety scoring may have limited the capacity to detect differential treatment effects. We note that the baseline values for several psychological measures were numerically higher in the placebo group; although such imbalances can arise by chance in randomized designs, particularly with multiple correlated outcomes, this pattern may increase susceptibility to regression-to-the-mean effects and should be considered when interpreting the results. Importantly, the study was conducted and analysed according to a pre-specified statistical analysis plan, consistent with established standards for RCTs. The introduction of additional covariate adjustments that were not defined a priori would represent a post hoc modification of the analytical approach, which may increase the risk of bias and potentially compromise the internal validity of the findings.

Future research should include participants with higher baseline symptom burden and biomarkers to explore mechanisms, utilize cross-over or enriched designs to reduce placebo impact and evaluate long-term maintenance of effects. Additionally dietary monitoring should be assessed through the trial to ensure that microbiome changes over time are not influenced by changes in diet between the groups.

## 5. Conclusions

This study adds to the growing literature examining the potential role of probiotics and postbiotics in mental wellbeing. Both the probiotic and postbiotic blends were shown to be well tolerated. Whilst the primary outcome was not met, both probiotic and postbiotic interventions resulted in improved vitality scores, with the postbiotic showing beneficial changes in perceived stress scores. Moreover, microbiome analysis revealed an inverse correlation of *Bifidobacterium longum* abundance with the total PSS total score. Thus, the study findings support the need for further adequately powered trials into the targeted application of these probiotic and postbiotic blends in healthy subclinical populations to improve mental health outcomes.

## Figures and Tables

**Table 1:** Baseline characteristics: Part I.

Characteristic	Overall (*n* = 96)	Probiotic (*n* = 48)	Placebo (*n* = 48)	*p*-Value
Age (years)	35.0 ± 12.0	34.0 ± 11.0	35.0 ± 12.0	0.714
Range	18–62	18–58	19–62	—
BMI (kg/m^2^)	27.0 ± 5.7	28.0 ± 5.3	26.1 ± 6.0	0.017
Range	18.6–44.2	18.6–40.4	19.3–44.2	—
Total body weight (kg)	79.0 ± 19.0	82.0 ± 17.0	76.0 ± 20.0	0.014
Range	51–129	51–124	53–129	—
Standard drinks/week	3.79 ± 3.04	4.26 ± 3.12	3.30 ± 2.91	0.082
Range	1–15	1–14	1–15	—
HAM-A Total Score	13.0 ± 7.0	12.0 ± 7.0	14.0 ± 8.0	0.504
BDI Total Score	12.0 ± 6.0	11.0 ± 6.0	13.0 ± 6.0	0.200
PHQ-9 Total Score	5.3 ± 3.0	5.0 ± 2.9	5.6 ± 3.1	0.300
STAI-State	42.0 ± 9.0	40.0 ± 8.0	43.0 ± 9.0	0.051
STAI-Trait	46.0 ± 9.0	45.0 ± 9.0	47.0 ± 8.0	0.300
PSS Total Score	18.9 ± 5.3	18.1 ± 5.6	19.8 ± 4.8	0.110
GSRS Total Score	1.66 ± 0.59	1.65 ± 0.58	1.68 ± 0.62	0.852
Reflux	1.43 ± 0.88	1.54 ± 1.00	1.32 ± 0.73	0.434
Abdominal Pain	1.78 ± 0.73	1.72 ± 0.73	1.84 ± 0.74	0.376
Indigestion	1.88 ± 0.86	1.83 ± 0.76	1.93 ± 0.97	0.869
Diarrhea	1.46 ± 0.65	1.48 ± 0.64	1.45 ± 0.67	0.593
Constipation	1.08 ± 0.59	1.03 ± 0.50	1.13 ± 0.67	0.582

Data are represented as means ± SEM.

**Table 2:** Baseline characteristics: Part II.

Characteristic	(*n* = 8)
Age (years)	39 (16)
Range	21–62
Female	7/8 (88%)
Male	1/8 (13%)
Ethnicity, white Irish	8/8 (100%)
Non-smoker	8/8 (100%)
Alcohol consumption	6/8 (75%)
Standard drinks/week	2.67 (1.63)
Range	1.00–5.00
Total body weight (kg)	69 (11)
Range	56–85
BMI (kg/m^2^)	25.30 (2.95)
Range	21.84–30.85

## Data Availability

The data presented in this study are available on request from the corresponding author due to privacy.

## Supplementary Materials

The following supporting information can be downloaded at: https://www.mdpi.com/article/10.3390/brainsci16040419/s1, Table S1: Part I participant assessment scores. Table S2: Part II participant assessment scores. Figure S1: HAM-A scoring analysis from Part II over 6 weeks. Figure S2: Assessment of sleep quality and gastrointestinal symptoms in participants from Part I over 12 weeks. Figure S3: Mean relative abundances per group of the seventy most abundant (a) genera and (b) Species. Label ‘Others’ includes the rest of the genera. Figure S4: Microbiome diversity analysis and PCoA plot of beta diversity.

## Abbreviations

The following abbreviations are used in this manuscript:

HAM-A	Hamilton Anxiety Rating Scale
BDI	Beck Depression Inventory
PHQ-9	Patient Health Questionnaire-9
PSS	Perceived Stress Scale
STAI	State-Trait Anxiety Inventory
PSQI	Pittsburgh Sleep Quality Index
SF-36	36-Item Short-Form survey
GSRS	Gastrointestinal Symptom Rating Scale
SCFAs	Short-Chain Fatty Acids
RCTs	Randomized Clinical Trials
HPA	Hypothalamus–Pituitary–Adrenal
SAP	Statistical Analysis Plan
AEs	Adverse Events
ITT	Intention To Treat
BMI	Body Mass Index
QoL	Quality of Life
PCoA	Principal Coordinate Analysis
NES	Normalized Enrichment Score
KEGG	Kyoto Encyclopedia of Genes and Genomes
GSEA	Gene Set Enrichment Analysis
BDNF	Brain-Derived Neurotrophic Factor
