# Supplementary material for: A Randomised, Double-Blind, Placebo-Controlled Trial of Probiotic and Postbiotic Strains in Healthy Adults with Self-Reported Anxiety: Effects on Mood, Vitality, Quality of Life and Perceived Stress
Source: Brain Sci. 2026 Apr 16;16(4):419. doi: 10.3390/brainsci16040419 (PMC13114223; doi:10.3390/brainsci16040419)
Supplement: Supplementary file 1 [file brainsci-16-00419-s001.zip › brainsci-4073777-supplementary.pdf]

Supplementary table S1. Part I participant assessment scores

	Placebo (*n*=48)	Probiotic (*n*=48)	*p*
**HAM-A**			
Week 0	13.67 (1.09)	12.25 (0.99)	
Week 12	7.17 (0.69)	7.06 (0.72)	0.761
**BDI**			
Week 0	13.13 (0.84)	11.40 (0.89)	
Week 12	7.98 (0.85)	6.30 (0.71)	0.274
**STAI-T**			
Week 0	46.92 (1.22)	45.08 (1.28)	
Week 12	39.29 (1.26)	37.62 (1.30)	0.647
**STAI-A**			
Week 0	43.46 (1.30)	40.04 (1.15)	
Week 12	35.96 (1.26)	32.72 (1.35)	0.356
**PHQ-9**			
Week 0	5.64 (0.45)	5.00 (0.42)	
Week 12	3.66 (0.38)	2.65 (0.31)	0.089
**PSS**			
Week 0	19.79 (0.70)	18.06 (0.81)	
Week 12	15.43 (0.76)	12.94 (0.88)	0.122
**Cortisol (C_{max})**			
Week 0	23.73 (1.59)	19.85 (1.60)	
Week 12	17.80 (1.10)	20.40 (2.24)	0.039
**PSQI**			
Week 0	7.29 (0.39)	7.02 (0.43)	
Week 12	6.10 (0.35)	5.98 (0.36)	0.971
**GSRS**			
Week 0	1.68 (0.09)	1.65 (0.08)	
Week 12	1.54 (0.08)	1.50 (0.08)	0.611
**SF-36 (PCS)**			
Week 0	55.83 (1.01)	57.04 (0.68)	
Week 12	56.62 (0.84)	56.79 (0.62)	0.751
**SF-36 (MCS)**			
Week 0	40.44 (1.11)	41.34 (1.17)	
Week 12	47.62 (0.94)	49.76 (1.13)	0.158
**SF-36 (Vitality)**			
Week 0	44.92 (1.28)	45.73 (1.32)	
Week 4	46.91 (1.20)	49.81 (1.35)	
Week 6	47.42 (1.19)	51.92 (1.16)	
Week 12	50.06 (1.12)	51.90 (1.13)	0.017

HAM-A, Hamilton anxiety rating scale; BDI, Beck's Depression Inventory; STAI, State/Trait Anxiety Inventory (-T: trait; -S: state); PHQ-9, Patient Health Questionnaire-9; PSS, Perceived Stress Scale; PSQI, Pittsburgh Sleep Quality Index; GSRS, Gastrointestinal Symptom Rating Scale; SF-36, Short-Form 36 health survey; PCS, Principle component summary; MCS. Mental Component Summary. Data are presented as means±(SEM). ANCOVA were performed with the groups (placebo, probiotic) as a between-subjects factor and time points as a within groups factor. The *p* values displayed represent the group x time interaction effects.

Supplementary table S2. Part II participant assessment scores

	Placebo (*n*=8)	Postbiotic (*n*=8)	*p*
**HAM-A**			
Week 0	15.63 (2.60)	14.50 (2.87)	
Week 6	12.88 (2.72)	10.75 (2.88)	1.000
**BDI**			
Week 0	16.25 (1.11)	16.88 (1.30)	
Week 6	12.75 (2.00)	12.63 (2.40)	0.572
**STAI-T**			
Week 0	47.38 (3.02)	49.13 (1.94)	
Week 6	44.13 (2.79)	45.63 (3.28)	0.889
**STAI-A**			
Week 0	43.88 (3.43)	47.50 (3.17)	
Week 6	39.50 (4.51)	43.25 (3.70)	0.984
**PHQ-9**			
Week 0	6.63 (0.53)	6.75 (0.88)	
Week 6	5.25 (1.28)	5.38 (1.27)	1.000
**PSS**			
Week 0	22.50 (0.91)	22.00 (0.71)	
Week 6	20.25 (1.01)	17.50 (1.92)	0.404
**Cortisol (C_{max})**			
Week 0	23.67 (4.21)	18.66 (3.59)	
Week 6	22.77 (3.01)	20.35 (3.12)	0.458
**PSQI**			
Week 0	9.88 (0.61)	10.50 (0.63)	
Week 6	9.13 (0.44)	8.50 (1.09)	0.170
**GSRS**			
Week 0	2.11 (0.20)	2.08 (0.22)	
Week 6	1.94 (0.21)	1.69 (0.21)	0.260
**SF-36 (PCS)**			
Week 0	59.95 (1.66)	56.58 (1.84)	
Week 6	56.71 (2.60)	57.06 (2.04)	0.091
**SF-36 (MCS)**			
Week 0	36.48 (2.30)	38.70 (2.19)	
Week 6	41.43 (3.51)	45.65 (3.77)	0.484
**SF-36 (Vitality)**			
Week 0	42.57 (3.07)	41.09 (2.48)	
Week 6	44.06 (3.91)	49.26 (2.72)	0.017
**SF-36 (SF)**			
Week 0	41.67 (2.75)	39.17 (3.13)	
Week 6	45.44 (3.13)	49.82 (2.68)	0.010

Classification: Confidential

HAM-A, Hamilton anxiety rating scale; BDI, Beck's Depression Inventory; STAI, State/Trait Anxiety Inventory (-T: trait; -S: state); PHQ-9, Patient Health Questionnaire-9; PSS, Perceived Stress Scale; PSQI, Pittsburgh Sleep Quality Index; GSRS, Gastrointestinal Symptom Rating Scale; SF-36, Short-Form 36 health survey; PCS, Principle component summary; MCS. Mental Component Summary; SF, Social Functioning. Data are presented as means±(SEM). RM ANOVA were performed with the groups (placebo, probiotic) as a within-subjects factor and time points as a within groups factor. The *p* values displayed represent the group x time interaction effects.

Supplementary Figure S1.

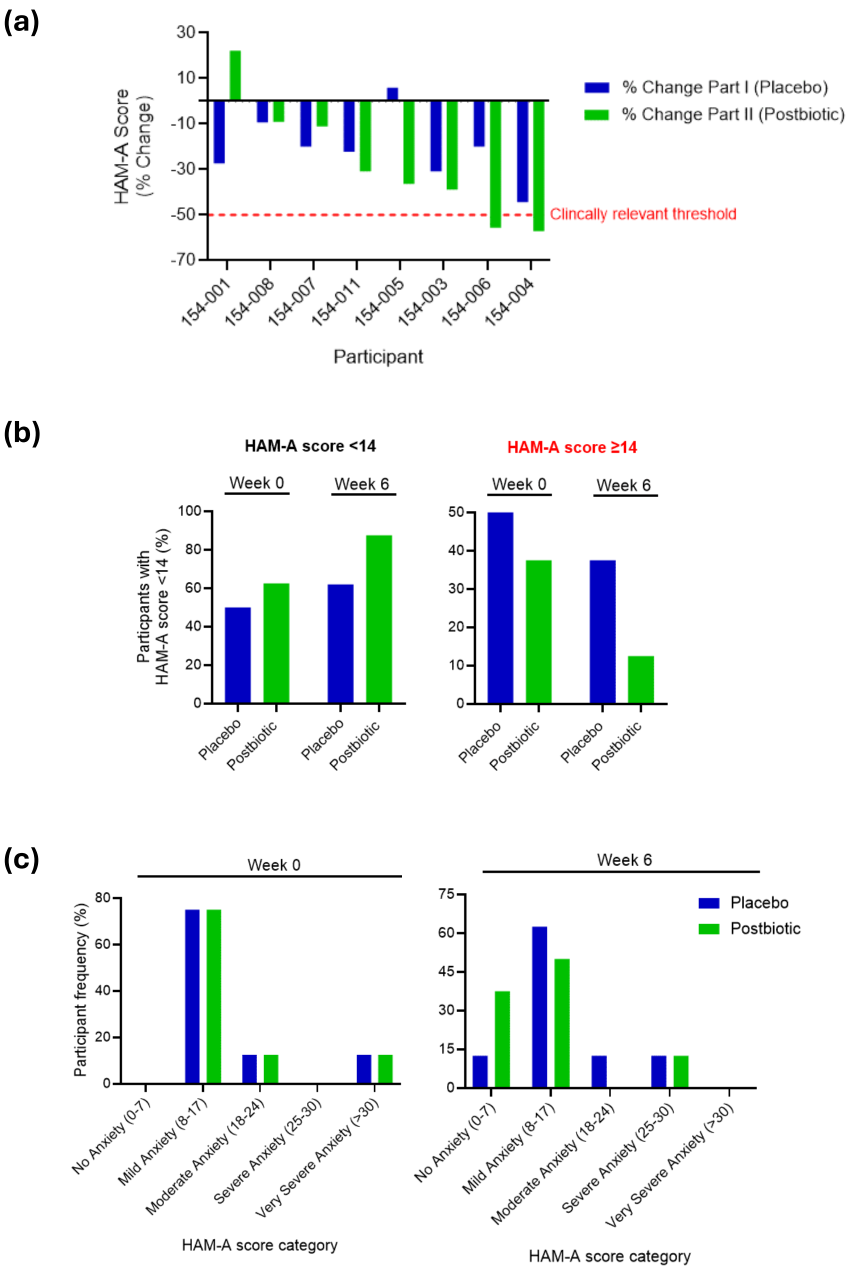

Supp. Figure S1. HAM-A scoring analysis from part II over 6 weeks**. (a)** Percent change in HAM-A scores from week 0 to week 6 plotted per participant for both placebo (part I) and postbiotic phases. Dashed red line indicates the clinical relevant threshold (≥50% reduction). **(b)** Frequency of participants in both placebo and postbiotic phases which obtained a HAM-A score <14 (left panel) and ≥14 (right panel) after 6 weeks (*n*=8). **(c)** Assessment of HAM-A scoring by category. The frequency of participants per HAM-A category for both placebo and postbiotic phases were assessed at week 0 (left panel) and after 6 weeks (right panel).

Supplementary Figure S2.

(a)

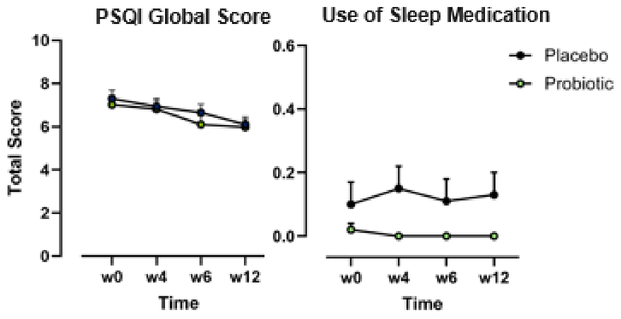

(b)

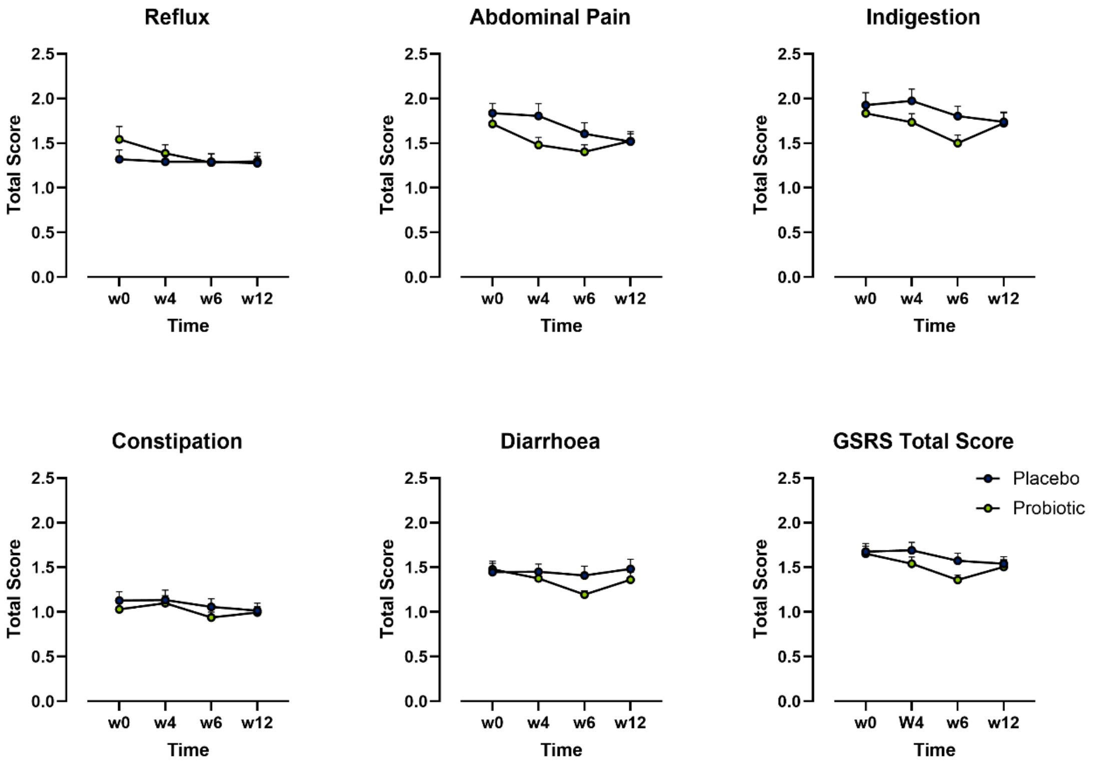

Supp. Figure S2. Assessment of sleep quality and gastrointestinal symptoms in participants from part I over 12 weeks**. (a)** Global score of the Pittsburgh Sleep Quality Index (PSQI; left panel) and use of sleep medication score (right panel) for participants in both placebo (*n*=48) and probiotic (*n*=48) arms. A borderline reduction in sleep medication usage (ANCOVA *p=0.067)* over 12 weeks was observed in the probiotic arm. **(b)** Participants in both placebo (*n*=48) and probiotic (*n*=48) arms scored various gastrointestinal symptoms over 12 weeks using the gastrointestinal symptoms rating scale (GSRS) ranging from *1* (no discomfort)-*7* (severe discomfort). Data points show the mean score. Error bars represent SEM.

Supplementary Figure S3.

(a)

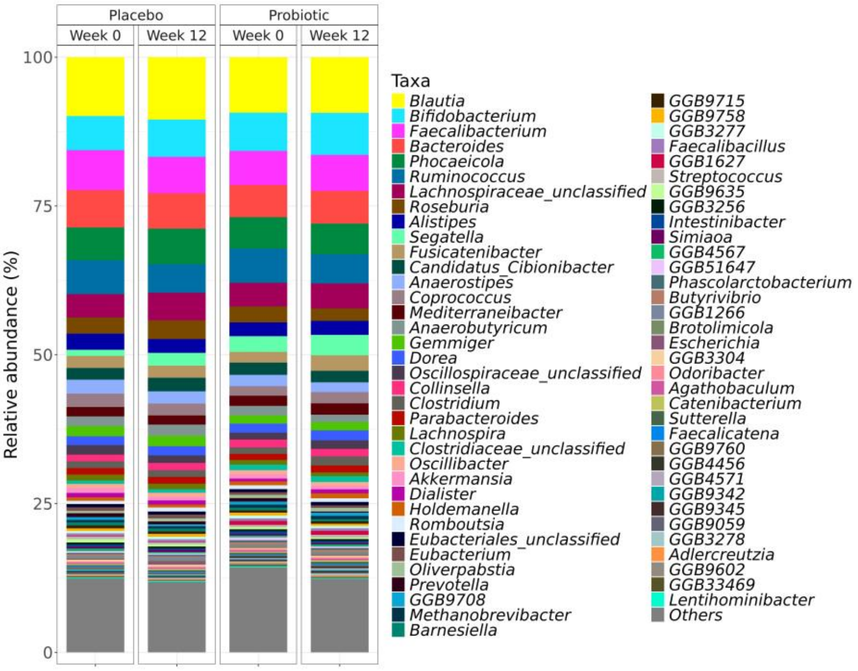

(b)

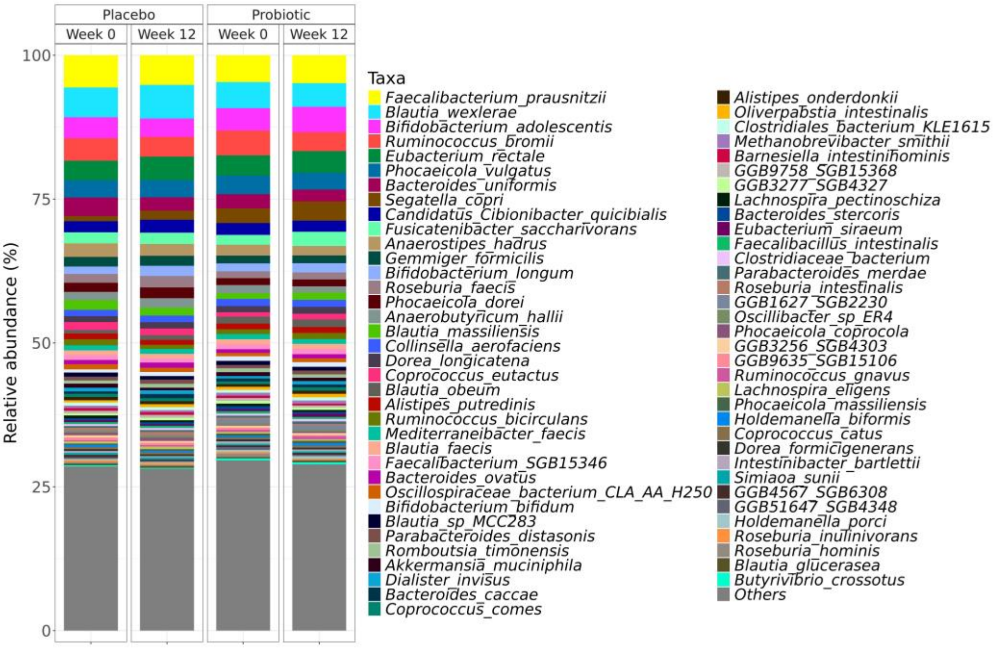

Supp. Figure S3. Mean relative abundances per group of the seventy most abundant **(a)** genera and **(b)** Species. Label 'Others' includes the rest of the genera.

Supplementary Figure 4.

(a)

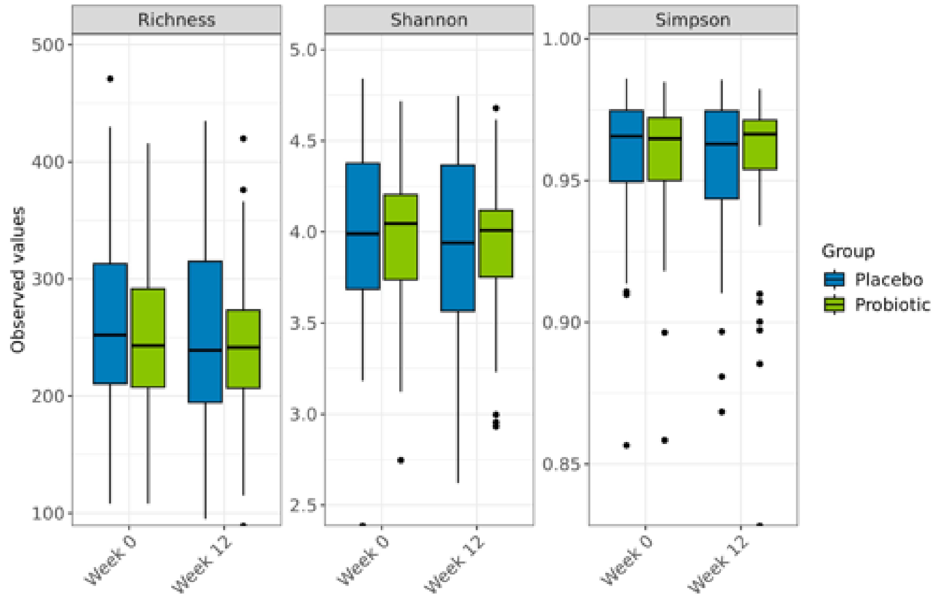

(b)

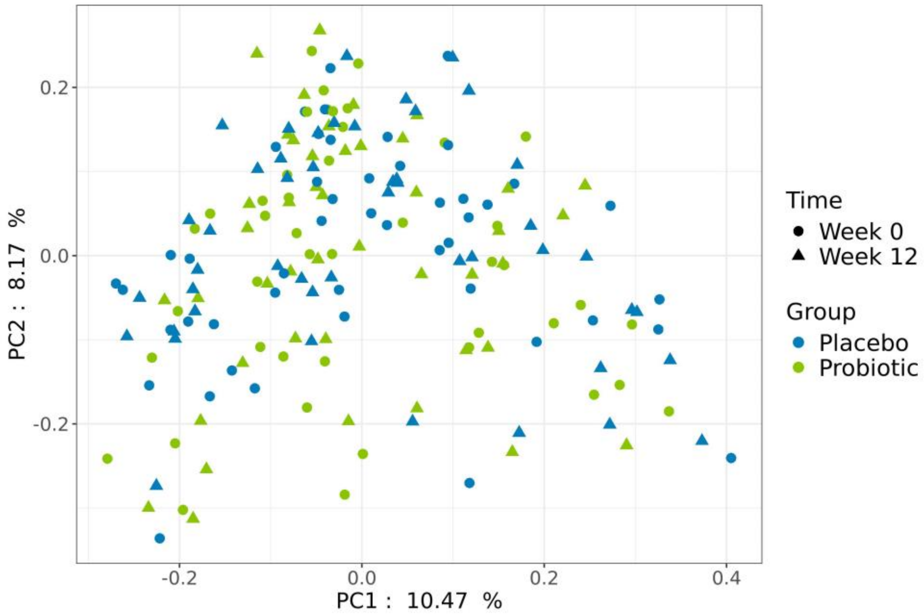

Supp. Figure S4. (a) Boxplots of Richness, Shannon and Simpson indexes in Placebo and Probiotic groups over Timepoints Week 0 and Week 12 on species. Wilcoxon test was applied between times on each group and between groups on each time, with no significant results. **(b)** PCoA plot of beta diversity based on Bray Curtis distance analysis in different groups and times. Each dot represents a sample and includes the ID of its Subject.
